# A New Skin Manifestation at the Site of a Previously Healed Dermatosis: A Case of Wolf’s Isotopic Response

**DOI:** 10.7759/cureus.11381

**Published:** 2020-11-08

**Authors:** Syed Munir, Dania Abu-Jubara, Musa Abu-Jubara, Carla Antypas, Cameron Petro-Sakuma

**Affiliations:** 1 Internal Medicine, Arkansas College of Osteopathic Medicine, Fort Smith, USA; 2 Emergency Medicine, Arkansas College of Osteopathic Medicine, Fort Smith, USA; 3 Family Medicine, Arkansas College of Osteopathic Medicine, Fort Smith, USA; 4 Internal Medicine, Unity Health, Searcy, USA

**Keywords:** shingle skin rash, herpes zoster virus, wolf’s isotopic response, furunculosis, post-herpetic isotopic response, postherpetic neuralgia, dermatosis

## Abstract

Wolf’s isotopic response (WIR) is an uncommon phenomenon that refers to the occurrence of a new skin condition at the location of a previously healed dermatosis. We describe an unusual manifestation of bacterial furunculosis which arose as an isotopic response following a herpes zoster episode. The initial skin disease in most cases is herpes zoster and the isotopic response is a granulomatous reaction.

A 65-year-old female with a history of chronic lymphocytic leukemia (CLL) and currently on chemotherapy regimen presented with a pustular skin rash on the posterior scalp extending to the posterior right neck and shoulder. Prior to this presentation, the patient was treated for three weeks with valacyclovir for herpes zoster infection which improved her skin condition. During the current hospitalization, the patient’s wound cultures from the pustule revealed the growth of methicillin-resistant *Staphylococcus aureus* (MRSA). Although the patient was on immunosuppressive therapy, her white blood cell (WBC) count increased to 9.9 x 10^3^/μL. After receiving vancomycin and valacyclovir, her cutaneous condition eventually improved. She was transitioned to oral clindamycin and discharged to a rehabilitation facility.

This case describes an immunocompromised patient who was treated for herpes zoster, improved after treatment, and then developed MRSA furunculosis at the same site. It is of significance to report such manifestations, especially in immunocompromised patients, as it could be underdiagnosed. It is also important to inquire about the patients’ herpes-related medical history because herpes is the most common initial dermatosis reported in the literature. In such cases of suspected WIR, it is vital to obtain a biopsy before starting treatment with antiviral medication to rule out the possibility of malignancy.

## Introduction

Wolf’s isotopic response (WIR) refers to the occurrence of a new skin condition that appears in the same location (isotopic) as a previously healed and unrelated skin condition. Even though WIR was first mentioned in 1955, the phenomenon remains rarely diagnosed. It is pertinent to note the subtle difference between WIR and Koebner’s isomorphic response. Koebner’s response describes the appearance of an existing skin lesion with similar morphology arising at sites of skin injury, isomorphic [1]. A wide range of predisposing factors have been reported to present as WIR, but recent literature has shown increased reported cases with an underlying herpes infection, as suspected in our patient [2]. If the isotopic response follows a herpetic infection the term post-herpetic isotopic response (PHIR) is used [3]. We describe a case of PHIR manifesting as furunculosis.

## Case presentation

**Figure 1:**
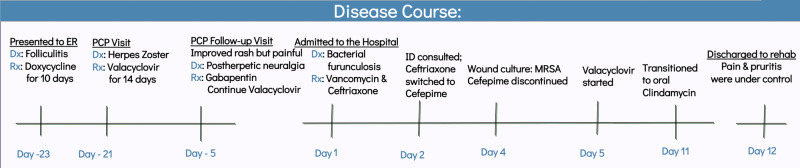
Disease course. Dx, diagnosis; Rx, prescription medication; PCP, primary care provider; ID, infectious disease; MRSA, methicillin-resistant *Staphylococcus aureus*

A 65-year-old Caucasian female presented with a three-week history of a moderately painful erythematous and pustular skin rash on the posterior head, extending to the posterior neck, right ear, and right shoulder (Figure 1). She has a past medical history of childhood chickenpox and current chronic lymphocytic leukemia (CLL) of B cell type which was last treated six-weeks ago with a chemotherapy regimen, bendamustine and rituximab.

The patient was given a 10-day course of doxycycline after being seen in the ED three-weeks prior to arrival. Two days later, her primary care provider diagnosed her with herpes zoster virus (HZV) due to its dermatological distribution. She was given a 14-day course of valacyclovir (1 g, three times a day) and instructed to continue (500 mg, twice per day) for prophylaxis. Two weeks later, via telemedicine, there was an improvement in the appearance of her rash but she reported pain on the right side of her neck. A timeline of her disease course is shown in Figure 1. 

**Figure 2:**
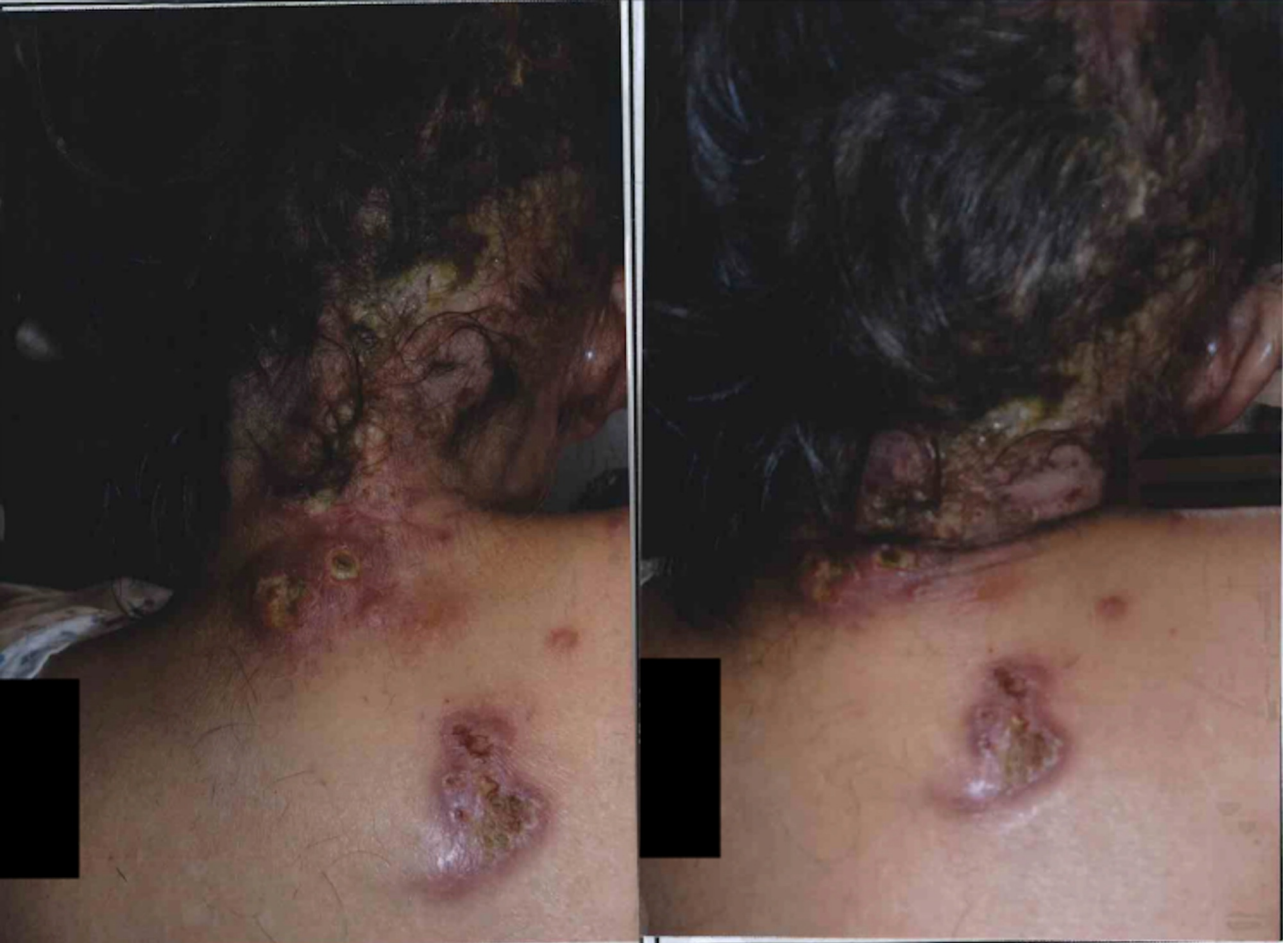
Lesions upon admission to the hospital.

Five days later, the patient presented to the ED due to a new pustular eruption along upper cervical dermatomes on the posterior neck and right shoulder indicating multiple sensory ganglia involvement (Figure 2). She noted that the rash was pruritic, painful, and easily aggravated by light touch and air. Her auditory and visual sensations were intact and no facial paralysis was observed. The diagnosis of bacterial furunculosis was made.

**Figure 3:**
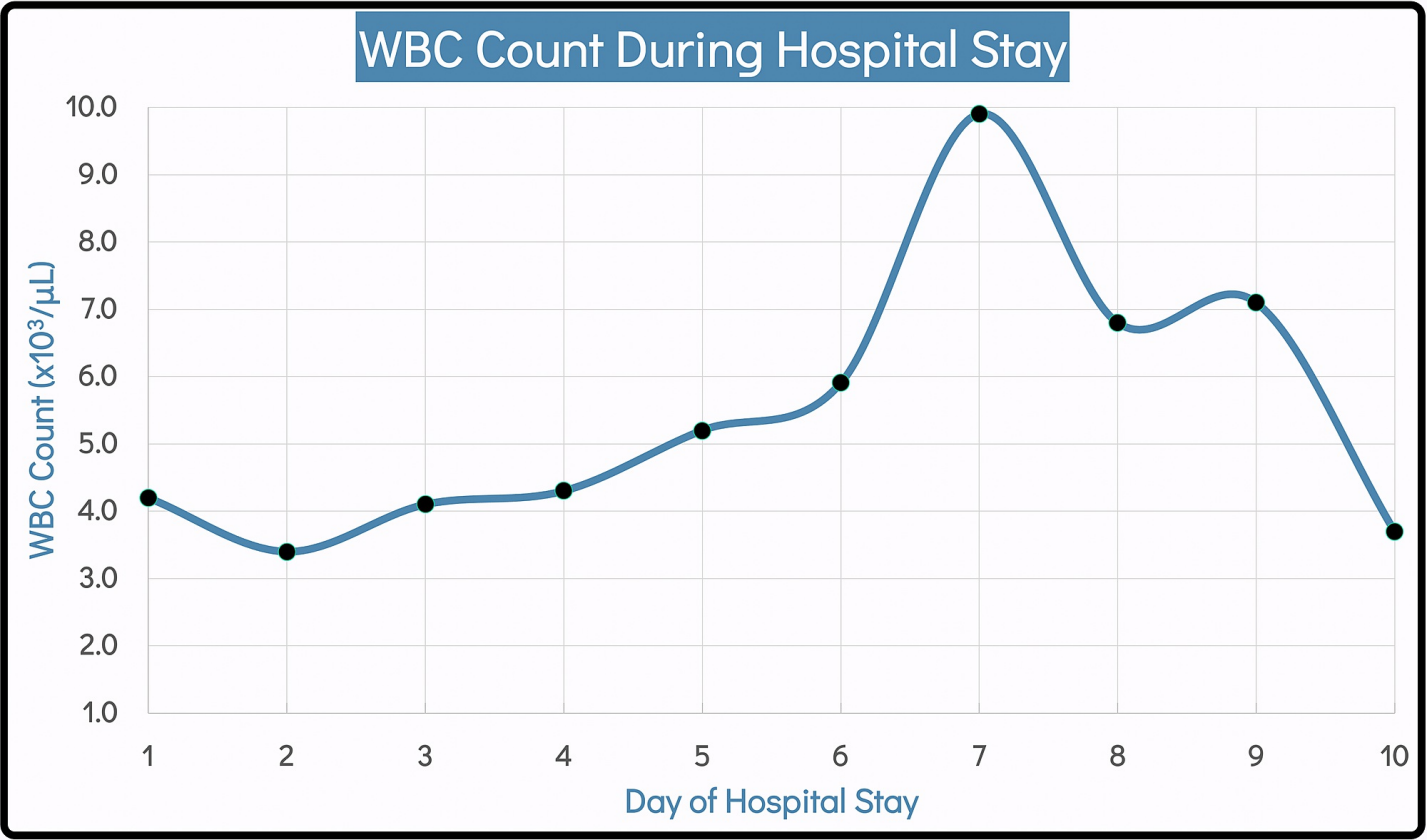
WBC count during the hospital stay. WBC, white blood cell

The patient was admitted to the inpatient service and was started on vancomycin, ceftriaxone, morphine, and diphenhydramine. Allodynia and pruritus may be severe in some presentations, which can be explained by WIR having involvement with the peripheral sensory fibers [4]. Moreover, her white blood cell count was 4.2 x 103/μL, which was monitored during her hospital stay (Figure 3). A CT scan of the head and cervical spine, and X-ray of the chest showed no acute findings. The oncology and infectious disease specialists were consulted. The oncologist recommended transfusing the patient with one unit packed red blood cells if her hemoglobin fell below 8.0 g/dL. This was done once at the beginning of her hospital stay. The infectious disease physician suggested switching ceftriaxone to cefepime. However, the cefepime was later discontinued after wound cultures grew methicillin-resistant *Staphylococcus aureus* (MRSA), and vancomycin was continued.

On day six of her hospital stay, the patient reported a sudden worsening of her pain. She was started on valacyclovir (1 g, twice a day). A brain MRI without contrast showed concern for possible inflammation of the right facial nerve. The suspicion for Ramsay Hunt syndrome (RHS) (herpes zoster oticus) led to a consultation with an otolaryngologist, ruling out the diagnosis due to no evidence of facial nerve paralysis.

**Figure 4:**
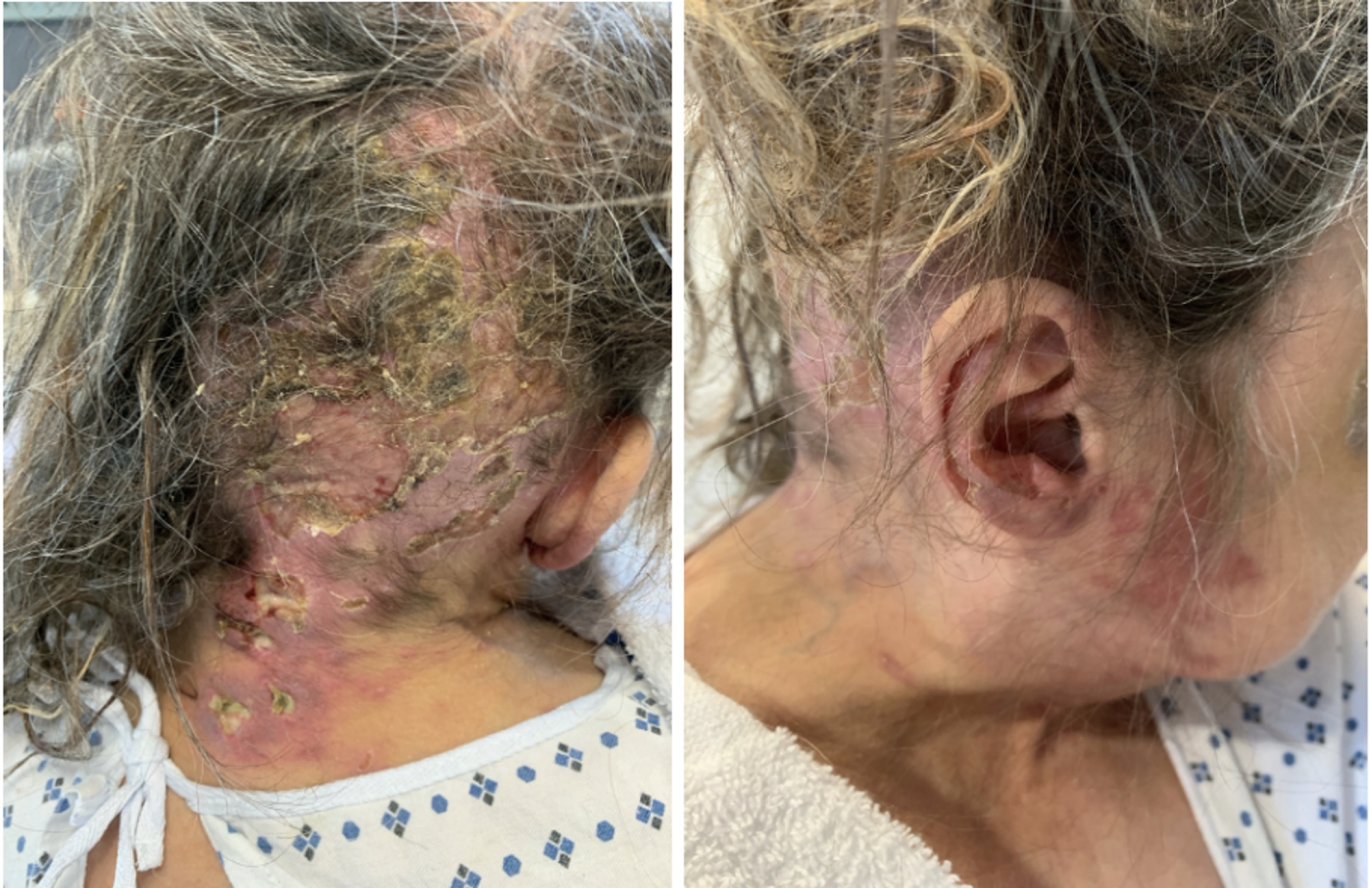
Lesions upon discharge from the hospital.

Eventually, the patient’s rash appeared to be improving and no purulent drainage was observed (Figure 4). Given her older age, complex history, and no complete resolution of her skin condition, the patient was discharged to a long-term acute care facility and her medications were transitioned from intravenous to oral route. She was instructed to continue valacyclovir for prophylaxis.

## Discussion

Herpes zoster virus is caused by the reactivation of varicella zoster virus (VZV) that develops more commonly in individuals older than 50 years of age and immunocompromised patients due to decreased T cell-mediated immunity. It typically begins as an erythematous papular rash, usually in a single or contiguous dermatomal pattern spanning several areas eventually becoming ulcerative. In older and immunocompromised patients, a hemorrhagic rash may even develop. Due to the complex nature of our patient’s presentation, we had a lengthy differential diagnosis list. Several herpes-related complications were considered, including postherpetic neuralgia, herpes zoster oticus (RHS), herpes zoster ophthalmicus, acute retinal necrosis, bacterial infections, Koebner’s isomorphic response, and WIR.

Herpes zoster ophthalmicus was ruled out as no ophthalmic lesions or visual disturbances were observed. RHS was initially considered due to the presence of CN VII inflammation on the MRI scan. The patient’s facial nerve was clinically intact and her external auditory meatus was clear, which made the otolaryngologist rule it out. Moreover, cervical dermatomes’ involvement is quite rare in classic RHS [5]. Trigeminal neuralgia was also suspected as the patient complained of severe sharp, lightning-like pain along the mandibular branch of the trigeminal nerve. Since the pain was constant especially in other dermatomal regions alternative diagnoses were sought out. Postherpetic neuralgia was likely as it commonly presents as a significant pain persisting for 90 days after the onset of rash. However, the pain was not her only complaint. Given the new onset of the bacterial furunculosis, further diagnoses were explored. 

Wolf’s isotopic response refers to the occurrence of a new skin condition at the site of a previously healed unrelated skin condition [6]. In this case, the isotopic response manifested as furunculosis. The most common predisposing skin disease is VZV or HZV infection as seen in our patient [7]. PHIR is the term used if the isotopic response is to follow a herpetic infection. While not yet fully understood, multiple theories and evidence suggest that the initial dermatosis might be a result of several factors including viral, vascular, immune, and neural elements [1]. The most favored theory regarding its pathogenesis suggests that WIR develops from disrupted and suppressed immunity, secondary to cutaneous nerve damage [4]. HZV infection injures cutaneous nerves and elicits the release of mediators such as calcitonin gene-related peptide and substance P [8]. This results in a localized impairment of immunity. Therefore, the comparable pathophysiology between WIR and HZV proposes a possible link between the two conditions. The latency and time intervals between primary and secondary dermatosis can range from a few weeks to months, to years which may be due to a type III or IV hypersensitivity reactions [4, 9]. A variety of skin conditions have been reported to present at the initial site of healed HZV such as lichenoid dermatitis, granulomatous reactions, skin tumors, psoriasis, and infections [10]. Recent literature reveals more cases presenting with underlying malignancies [9-10]. Although a biopsy was not obtained in this case, its indication could help to identify the histopathological pattern of the second dermatosis as well as to rule out the infiltration of hematologic malignancies [10-11].

## Conclusions

While WIR may be lacking in incidence, the rarity could be due to its rate of underdiagnosis. However, it is important to understand the pathogenesis of WIR in order to have an index of suspicion when a patient presents with it and thereby, appropriately diagnosing and managing it. Moreover, a focused history is needed to inquire about malignancies and any past herpes-related dermatological conditions. For a definitive diagnosis, a biopsy should be obtained before antiviral medication to rule out underlying malignancies. Lastly, effective treatment will be based on rapid intervention and appropriate symptomatic management. 
